# Conversion of Verbal Response Scales: Robustness Across Demographic Categories

**DOI:** 10.1007/s11205-015-0897-6

**Published:** 2015-02-21

**Authors:** Tineke DeJonge, Ruut Veenhoven, Linda Moonen, Wim Kalmijn, Jacqueline van Beuningen, Lidia Arends

**Affiliations:** 1 Erasmus Happiness Economics Research Organization, Erasmus University Rotterdam, Rotterdam, The Netherlands; 2 North-West University, Potchefstroom, South Africa; 3 Statistics Netherlands, Heerlen, The Netherlands; 4 Institute of Psychology, Erasmus University Rotterdam, Rotterdam, The Netherlands; 5 Biostatistics, Erasmus MC, Rotterdam, The Netherlands

**Keywords:** Comparability of rating scales, Beta distribution, Reference distribution, Demographic categories, Happiness, Satisfaction with life

## Abstract

Happiness and life satisfaction have traditionally been measured using verbal response scales, however, these verbal scales have not kept up with the present trend to use numerical response scales. A switch from a verbal scale to a numerical scale, however, causes a severe problem for trend analyses, due to the incomparability of the old and new measurements. The Reference Distribution Method is a method that has been developed recently to deal with this comparison problem. In this method use is made of a reference distribution based on responses to a numerical scale which is used to decide at which point verbally labelled response options transit from one state to another, for example from ‘happy’ to ‘very happy’. Next, for each wave of the time series in which the verbal scale is used, a population mean is estimated for the beta distribution that fits best to these transition points and the responses in this wave. These estimates are on a level that is comparable to that of the mean of the reference distribution and are appropriate for use in an extended time series based on the responses measured using a verbal and a numerical scale. In this paper we address the question of whether the transition points derived for the general population can be used for demographic categories to produce reliable, extended time series to monitor differences in trends among these categories. We conclude that this is possible and that it is not necessary to derive transition points for each demographic category separately.

## Introduction

A change from using a verbal to using a numerical scale to measure happiness and life satisfaction disrupts the trends for demographic categories, and these discontinuities due to a change in scales have to be fixed in such a way that trend analyses can be made in an accurate way based on an extended time series which includes responses measured using the old verbal scale and those measured using the new numerical scale.

### The Need for, and Consequence of, a Change from Using a Verbal Scale to Using a Numerical Scale

Happiness and life satisfaction have traditionally been measured using verbal response scales, however, these verbal scales have not kept up with the present trend to use numerical response scales. An increasing body of research (e.g. OECD 2013) states that numerical scales are more suitable for measuring such constructs, because use of numerical scale minimises categorisation effects, improves data analysis and produces more reliable data. Furthermore, numerical scales are especially suitable for telephone interviews, because they are time saving and prevent response-order effects (Scherpenzeel 1999). At present, 11- and 10-point scales, with the anchor points^1^ defined by verbal labels, are used most frequently to measure subjective well-being (Voorpostel et al. 2009; OECD 2013; Van Beuningen et al. 2014).

Changing from a verbal scale to a numerical scale leads to an instantaneous discontinuity in the existing time series. This causes a severe problem for trend analyses, due to incomparability of the old and new measurements, meaning that, with the introduction of the numerical scale, current trends in well-being across demographic categories cannot be continued for long time series unless the discontinuity caused by the change of the scale type can be overcome in a suitable way.

### The Reference Distribution Method and the Research Question

The Reference Distribution Method is a method that has been developed recently to deal with the kind of problem presented above (DeJonge et al. 2014a). This method relies heavily on the Continuum Approach of Kalmijn (2010, Ch. VI) which is a method that is used to estimate a mean and standard deviation in a population based on the beta distribution that fits best to the points, upper boundaries, on a continuum bounded by 0 and 10 at which response options from a verbal or numerical primary scale transit from one state to another, for example from ‘happy’ to ‘very happy’, combined with the frequency distribution of this primary scale.

In the Reference Distribution Method the Continuum Approach is applied to estimate a reference distribution based on the response on a discrete numerical scale. This reference distribution is then used to determine at which transition points the upper boundaries of the response options from a verbal scale are positioned. Next, the Continuum Approach is used to estimate a population mean for the beta distribution that fits best to the upper boundaries determined in the previous step. This is done for each wave of a time series. These estimates are comparable to the mean of the reference distribution and can be used to extend a time series and to combine the responses measured using the verbal and numerical scales. In the remainder of this paper we will refer to the transition points derived from the reference distribution as ‘reference boundaries’.

To date, in the development of the Reference Distribution Method the focus has been placed on the general population in nations and no distinction has been made between demographic groups within a nation. The research question we addressed in this paper is: Can the reference boundaries derived for the general population be used for demographic categories to produce reliable extended time series to monitor differences in trends among these categories?

### Plan of this Paper

We had data sets from Statistics Netherlands and the Eurobarometer at our disposal for different demographic categories which we could use to answer the research question. These data are described in Sect. 2. The Reference Distribution Method, which we explain in Sect. 3, provides the basis for all the results presented in this paper. We applied the Reference Distribution Method to estimate reference distributions and derive reference boundaries from these for different demographic categories and different verbal scales. We will elaborate on this in Sect. 4. Using the reference boundaries we found, we estimated population means and the means for all the demographic categories for each wave of a time series on a continuum from 0 to 10. We use these estimates to answer the research question in Sect. 5. We end with a discussion in Sect. 6 and the main conclusions in Sect. 7.

## Data

An application of the Reference Distribution Method for trend analyses in different demographic categories requires time series of the responses to each of these categories on a verbal scale and for one wave of measuring the same topic on a numerical scale. A set of time series based on survey items^2^ taken from Statistics Netherlands data and the Eurobarometer provided the data for our application.

### Happiness and Satisfaction with Life: Statistics Netherlands

Statistics Netherlands measured happiness^3^ and satisfaction^4^ with life using 5-point verbal response scales in the Permanent Onderzoek Leef Situatie^5^ (POLS) in the period 1997–2008 and in the Social Cohesion survey in 2009. On average the number of respondents to the survey items on these topics added up to approximately 8,300 per year. The question about happiness was formulated as “To what extent do you consider yourself a happy person?” and was rated on a verbal response scale with the options ‘Unhappy’, ‘Not very happy’, ‘Neither Happy nor unhappy’, ‘Happy’ and ‘Very happy’. For life satisfaction the question was phrased as “To what extent are you satisfied with the life you currently lead?”. Respondents could tick one of the response options labelled ‘Not very satisfied, ‘Fairly satisfied’, ‘Satisfied’, ‘Very satisfied’ and ‘Extraordinarily satisfied’.

In 2012 Statistics Netherlands included the questions on happiness and life satisfaction in an experiment with a split-half design for the Social Cohesion Survey (Van Beuningen et al. 2014). For this survey respondents were at random assigned to a group that had to rate the traditional 5-point verbal scales and a group that had to rate the same questions on a numerical 11-point response scales of which only the anchor points had verbal labels. The question on happiness with the 11-point numerical scale was phrased as: “On a scale from 0 to 10 can you indicate to what extent you consider yourself to be a happy person? A score of 0 refers to being completely unhappy and a score of 10 refers to being completely happy”. The question on life satisfaction with a numerical scale was worded as: “On a scale from 0 to 10 can you indicate to what extent you are satisfied with the life you currently lead? A score of 0 refers to being completely dissatisfied and a score of 10 to being completely satisfied”.

Data in the experiment were collected using a sequential mixed mode design. People were sent an invitation and two reminder letters asking them to fill out the questionnaire online. Those who did not respond to this invitation were interviewed by phone if a telephone number was available. When no telephone number was available people were interviewed face-to-face at their home.

The survey for the split-half experiment was distributed among respondents of 15 years and older, but for this paper only respondents 18 years and older were included in the analyses, which gave a total number of 7,641 respondents. Distinctions were made between gender, age, {18–25, 25–35, 35–45, 45–55, 55–65 and 65 and older}, and education, {low, middle and high}, to differentiate demographic categories of respondents. The frequency distributions for the two 5-point scales and the two 11-point scales for each of these categories are included in Tables 3, 4, 5 and 6 in Appendix 1.

### Satisfaction with Life: The Eurobarometer

The Eurobarometer is a series of public opinion surveys that have been conducted regularly in the member states of the European Union on behalf of the European Commission since 1973. The standard version of the Eurobarometer contains an item on life satisfaction with the leading question ‘On the whole how satisfied are you with the life you lead?’ and a 4-point verbal scale with response options ‘Not at all satisfied’, ‘Not very satisfied’, ‘Fairly satisfied’ and ‘Very satisfied’. The standard Eurobarometer has a spring wave and an autumn wave and each survey consists of approximately 1,000 face-to-face interviews per participating country. In the autumn of 2011, prior to the standard survey, version 76.2 of the Eurobarometer was launched, in which the item for life satisfaction had to be rated on a 10-point numerical scale, with the anchor points labelled ‘Very dissatisfied’ and ‘Very satisfied’ (European Commission 2012b).

Since we are interested in time series for life satisfaction, we selected only countries which had participated in the European Union for more than 10 years. In the fifteen European countries that joined the European Union before 2004, life satisfaction was measured for the entire period between 1997 and 2012 and also the item with the 10-point numerical scale was fielded in all these countries (Schmitt et al. 2008; European Commission, 2012a, b, 2013). The number of respondents to the Eurobarometer per country was too low to distinguish all the demographic categories we distinguished in the split-half experiment of Statistics Netherlands. Therefore, we divided the countries into two groups of countries:

- Northern Europe: Denmark, Sweden, Finland, Germany, Great Britain, The Netherlands, Belgium, France, Ireland, Luxemburg, Austria
- Southern Europe: Portugal, Spain, Italy and Greece

Then, for these two groups, we distinguished similar demographic categories to those used for the items of Statistics Netherlands. Only the three categories indicating the education level of a respondent are not fully comparable between the two datasets. We labelled these categories as ‘low’, ‘middle’ and ‘high’. In the Eurobarometer the education level classification is: in education until the age of 15, until the age between 16 and 19 years old and until an age of 20 years or over. The frequency distributions for the 5-point scale and the 10-point scale for each of these categories and the European regions can be found in Tables 7, 8, 9 and 10 of Appendix 2.

It is interesting to compare the frequencies with which the upper anchor points have been ticked in The Netherlands on the numerical scale of Statistics Netherlands scale and in Northern Europe on the numerical Eurobarometer scale. This frequency is more than three times higher in Northern Europe than in The Netherlands. This difference is rather due to the labelling of each of the anchor points than to a real difference in satisfaction: the wording ‘Completely satisfied’ used by Statistics Netherlands is likely to be interpreted differently from the wording ‘Very satisfied’ used in the Eurobarometer. Moreover the Northern Europeans ticked the anchor point more frequently than the preceding option labelled ‘9’. This phenomenon according to which the frequency with which respondents tick a ‘10’ stands out and is sometimes higher than the frequency by which a ‘9’ is ticked by respondents in the same sample is not unique as has been described by Brulé and Veenhoven (2014), who named this the ‘10-excess phenomenon’.

## The Reference Distribution Method

The idea that underlies the Reference Distribution Method is that the estimated means for equivalent questions about the same topic asked in different representative surveys in 1 year should be approximately the same when compared irrespective of the primary response scales used.

### Application of the Continuum Approach to Derive a Reference Distribution

In this section we will demonstrate an application of the Continuum Approach to derive a reference distribution. An outline of the Continuum Approach is given in Appendix 3. We assume that a discrete numerical scale with 10 or 11 points is available to derive a reference distribution. Furthermore, we assume that the upper boundaries of the response options of this scale are equidistant distributed over the 0–10 continuum. This assumption is a methodological choice (Kalmijn 2013), which provides a very useful basis for the Reference Distribution Method.

**Fig. 1:**
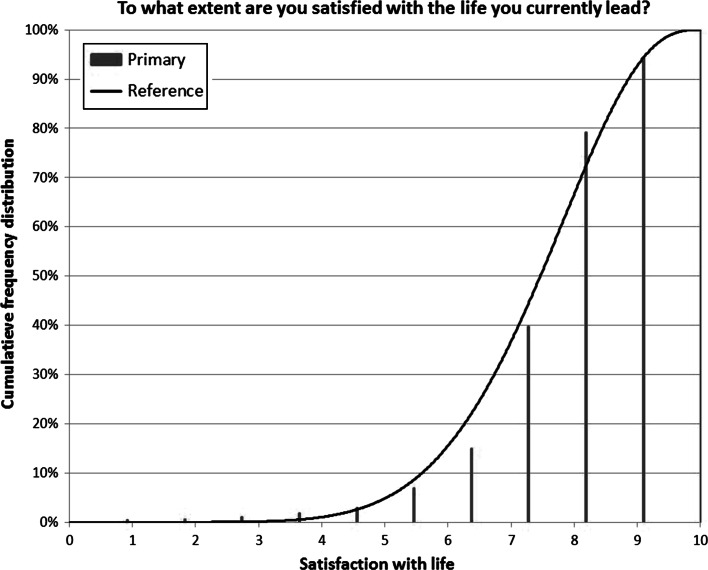
Best fitting beta distribution to life satisfaction measured on the numerical scale

How we applied the Continuum Approach to derive a reference distribution from the life satisfaction ratings on the numerical scale of the split-half experiment of Statistics Netherlands is shown in Fig. 1. In this figure the cumulative frequencies for life satisfaction are plotted as vertical bars at equal distances, starting at 0.91 for the response option at the lower end of the scale and ending with 10.0 for the option at the upper end of the scale. The best fitting beta distribution to the frequency distribution obtained by application of the Continuum Approach is depicted as a curve. It is clear that the beta distribution does not perfectly fit to the cumulative frequencies on the discrete numerical scale, but it is an approximation which underestimates the cumulative frequency at some points and overestimates it at other points.

The parameters of the beta distribution in Fig. 1 are *α* = 8.31 and *β* = 3.05, which, according to Eq. 3 in Appendix 3, corresponds to a mean of 7.31.

### Illustration of the Application of the Reference Distribution Method

An application of the Reference Distribution Method to derive reference boundaries for the response options of the verbal scale is explained in this section. The method itself is described in detail in DeJonge et al. (2014a). We applied the Reference Distribution Method to the reference distribution we found in the previous section for the verbal scale of the life satisfaction item of the split-half experiment and the ratings of the respondents of 18 years and older on this scale. The frequency distribution of these ratings was

- Extraordinarily satisfied: 7.2 %
- Very satisfied: 31.2 %
- Satisfied: 44.6 %
- Fairly satisfied: 11.1 %
- Not very satisfied: 5.9 %.

**Fig. 2:**
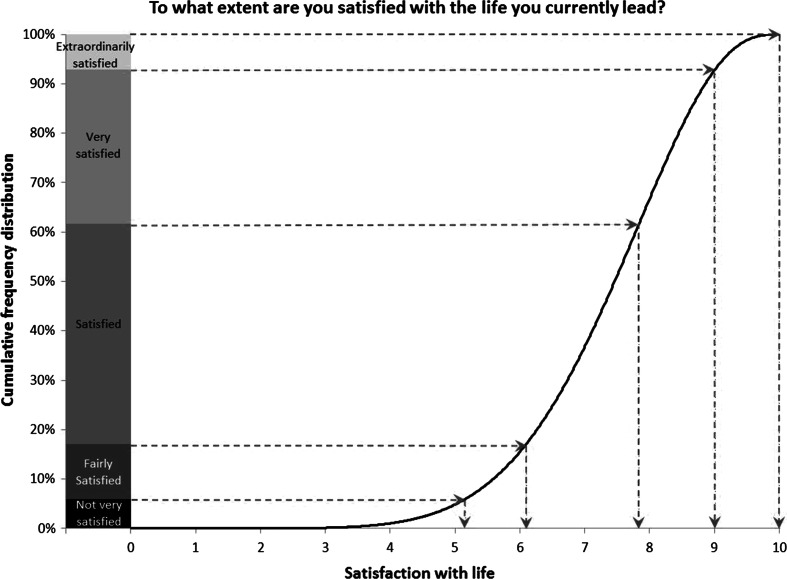
Illustration of the Reference Distribution Method

The corresponding cumulative frequency distribution is shown as a stack diagram on the left side of Fig. 2. The reference distribution is depicted to the right side of this cumulative frequency distribution.

The procedure to determine the reference boundaries between the response options of the primary verbal scale on the continuum from 0 to 10 is as follows, see also Fig. 2. For each response option, a horizontal line is drawn from the cumulative frequency displayed for the verbal scale to the point where it meets the reference distribution. At this point the value of the reference distribution is equal to the cumulative distribution on the verbal scale. A vertical line is drawn to the horizontal axis from this point downwards. The value at which the vertical line meets the horizontal axis is the position of the reference boundary of the corresponding response option. Following this procedure, the reference boundaries for the response options on the 0 to 10 continuum are consecutively 5.15, 6.09, 7.83, 9.00 and 10.00.

The reference boundaries and the cumulative frequencies measured on the verbal scale can in turn be used as input for the application of the Continuum Approach.

## Reference Boundaries in Different Demographic Categories

People will interpret verbal response options differently for all kinds of reasons such as personality, cultural context, demographics characteristics or the context of the scale. This makes it unlikely that the reference boundaries for the response options of a given scale for the general population are equal to those for subcategories with specific characteristics.

### Reference Boundaries and Estimated Means for Demographic Categories

To apply the Reference Distribution Method in an appropriate way, we had to adjust the verbal scale for happiness from Statistics Netherlands, because only a few of the respondents had ticked the option ‘unhappy’ in the split-half experiment. For the application of the reference distribution method such response options have to be combined with the preceding or succeeding option to obtain proper reference boundaries. Except for the age category from 55 to 64 years, in all demographic categories <1 % of the respondents selected this option as can be seen from Table 5 of Appendix 1. As a consequence, the application of the Reference Distribution Method will return a reference boundary equal to zero for this option for respondents aged from 35 to 44 years. Since the frequency with which the option ‘Not very happy’ was chosen was also small in all demographic categories, we combined the options ‘Unhappy’ and ‘Not very happy’ for the application of the Reference Distribution Method.

We applied the Reference Distribution Method to obtain reference boundaries for the verbal scales for life satisfaction of Statistics Netherlands and the Eurobarometer and the adjusted verbal scale for happiness of Statistics Netherlands. For each demographic category and each topic, either happiness or life satisfaction, we first determined the best fitting beta distribution to the cumulative frequencies measured on the corresponding numerical scale from the split-half experiment of Statistics Netherlands or Eurobarometer version 76.2 as we illustrated in Fig. 1. Next, we used these best fitting beta distributions to derive the reference boundaries between the response options of the verbal scales for each demographic category in the way we demonstrated in Fig. 2 based on the related cumulative frequency distribution measured in the split-half experiment of Statistics Netherlands or in version 76.3 of the Eurobarometer.

**Fig. 3:**
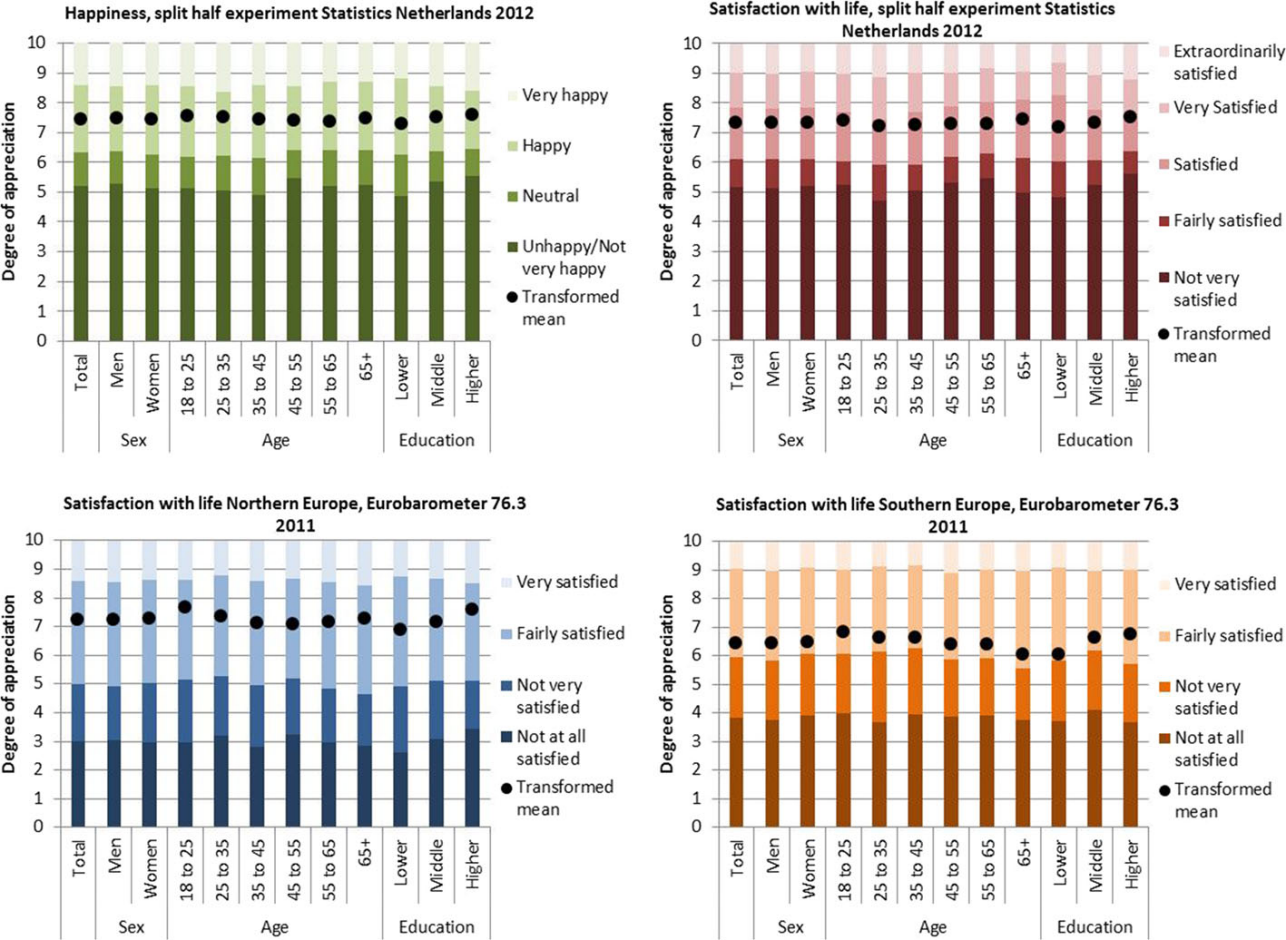
Reference boundaries and estimated means per demographic category

The results are displayed in Fig. 3 in which the response scales are visualized as vertical bars in which each interval represents the degree of appreciation expressed by a response option and in which the reference boundaries separate two adjacent response options. The estimated mean for a demographic category d in Fig. 3 is, in accordance with formula (3) in Appendix 3 equal to 10*α_d_/(α_d_ + β_d_), with α_d_ and β_d−_ the shape parameters of the beta distribution that fits best to the reference boundaries and the cumulative frequency distribution for the given demographic category.

From Fig. 3 it is obvious that differences in reference boundaries between demographic categories and the general population are most prominent for age categories and for categories based on the education level of the respondents. Apparently, gender does not make much of a difference, not in the split-half experiment or in the Eurobarometer.

The levels of the estimated means for Eurobarometer version 76.3 cannot be compared to those for life satisfaction in the split-half experiment, because the reference distribution in the split-half experiment was based on an 11-point numerical scale, whereas in the Eurobarometer a 10-point numerical scale was used.^6^ For this reason, the estimated means for the split-half experiment are visibly higher than those for the Eurobarometer. In addition, the anchor points of both numerical scales for life satisfaction were labelled differently.

The patterns of estimated means for the Eurobarometer are similar for Northern and Southern Europe, except that there is a substantial difference in the level: life satisfaction in Southern Europe is, in all demographic categories, lower than in Northern Europe. This comes with noticeably differences in reference boundaries between the two clusters of European countries.

As we stated above, the differences in boundaries can be attributed to a number of reasons. For the differences between age categories, it is imaginable that for example the response time of younger people is shorter than that of older people, whereas persons belonging to the latter category have a longer life experience than those who are younger. Both aspects may, among others, have an effect on the response patterns. The reasons for differences in boundaries however, fall outside the scope of this paper and have been addressed by others, see for example Hazelrigg and Hardy (2000), Storm et al. (1996), the National Research Council (2013), and DeJongeet al (2014b). We recall that the research question addressed in this paper is whether the reference boundaries derived for the general population can be used for demographic categories to produce reliable extended time series to monitor differences in trends between these categories.

### Differences in Estimated Means

A first step to answer the research question is to look at the differences in estimated means for demographic categories between the situation in which the reference boundaries for the general population are used and the situation in which category-specific boundaries are used. In Table 1 the estimated means as presented in Fig. 3 are given in the columns headed by ‘Reference boundaries category’. The estimated means based on the best fitting beta distributions to the reference boundaries for the general population and the cumulative frequencies measured in each demographic category are given in the columns headed ‘Reference boundaries population’.

**Table 1:** Differences in estimated means depending on reference boundaries used

Split-half experiment Statistics Netherlands 2012							
Category	Sub-category	Happiness			Satisfaction with life		
		Estimated means		Difference	Estimated means		Difference
		Reference boundaries category	Reference boundaries population		Reference boundaries category	Reference boundaries population	
Total	Total	7.45	7.45		7.32	7.32	
Sex	Men	7.46	7.44	0.02	7.32	7.34	−0.02
	Women	7.46	7.47	−0.01	7.32	7.30	0.02
Age	18–25	7.57	7.63	−0.06	7.40	7.47	−0.07
	25–35	7.53	7.71	−0.18	7.22	7.51	−0.29
	35–45	7.44	7.52	−0.08	7.27	7.37	−0.10
	45–55	7.40	7.36	0.04	7.30	7.23	0.07
	55–65	7.38	7.26	0.12	7.30	7.09	0.21
	65+	7.48	7.36	0.12	7.42	7.30	0.12
Edu-cation	Low	7.28	7.23	0.05	7.17	7.03	0.14
	Middle	7.51	7.49	0.02	7.31	7.35	−0.04
	High	7.60	7.68	−0.08	7.52	7.56	−0.04

Eurobarometer 2011 version 76.3							
Category	Sub-category	Satisfaction with life Northern Europe			Satisfaction with life Southern Europe		
		Estimated means		Difference	Estimated means		Difference
		Reference boundaries category	Reference boundaries population		Reference boundaries category	Reference boundaries population	
Total	Total	7.26	7.26		6.46	6.46	
Sex	Men	7.24	7.27	−0.03	6.43	6.54	−0.11
	Women	7.28	7.24	0.04	6.49	6.39	0.10
Age	18–25	7.66	7.60	0.06	6.84	6.78	0.06
	25–35	7.34	7.14	0.20	6.63	6.53	0.10
	35–45	7.13	7.17	−0.04	6.64	6.41	0.23
	45–55	7.09	6.97	0.12	6.41	6.49	−0.08
	55–65	7.18	7.26	−0.08	6.42	6.44	−0.02
	65+	7.28	7.47	−0.19	6.05	6.29	−0.24
Edu-cation	Low	6.91	6.90	0.01	6.07	6.15	−0.08
	Middle	7.16	7.06	0.10	6.66	6.54	0.12
	High	7.59	7.61	−0.02	6.78	6.92	−0.14

What is clear from Table 1 is that the differences between the estimated means obtained using the category-specific reference boundaries or by using the reference boundaries for the general population are small in general. Nevertheless, at least for some categories, there are differences. The difference is largest for satisfaction with life in the age category from 25 to 35 years of the split-half experiment. Using the reference boundaries for this category to estimate a mean for the best fitting beta distribution, would lead to a value which is almost 0.3 points lower than if the reference boundaries for the general population are used.

It would be premature to conclude on basis of Table 1 that category-specific reference boundaries should be used to estimate a mean for the demographic category. It very much depends on the purpose of the transition to the 0–10 continuum. If one is interested in the absolute difference in happiness or life satisfaction between demographic categories on a continuum from 0 to 10, than it seems evident to use category-specific reference boundaries. This is not obvious though, if one is interested, as we are given our research question, in whether or not trends in happiness and life satisfaction evolve in the same way in different demographic categories. In this case the absolute difference in happiness or life satisfaction is of less importance as long as the development of the trend in each category shows a reliable pattern after transition to the 0–10 continuum. From this perspective, to be able to answer the research question requires insight into the stability over time of the differences between the estimated means on the continuum for each demographic category obtained using the category-specific boundaries or the boundaries for the general population.

## Trends in Estimated Means in Different Demographic Categories

The evolution of happiness and life satisfaction over time may differ across demographic category. These differences in evolution have to be preserved after estimation of the corresponding means on a continuum from 0 to 10, independent of whether reference boundaries for the population of a whole are used or category-specific boundaries. If the latter is the case, then it suffices to apply the reference boundaries for the general population to obtain estimated means on a 0–10 continuum for monitoring differences in trends in extended time series.

### Trends in Estimated Means over Time for the General Population

We applied the Continuum Approach to the response of the general population to the items on happiness and life satisfaction for each wave of the surveys of Statistics Netherlands in the period 1997–2009 and to the item on life satisfaction for each wave of the Eurobarometer in the period 1997–2012. We made use of the reference boundaries for the general population for each wave, as shown in Fig. 3. In these periods there were no changes in the mode of surveying for both surveys (DeJonge et al. 2015). Because of that, the reference boundaries can be kept fixed over time and the differences in estimated means can solely be attributed to changes in the frequency distributions on the primary scale (DeJonge et al. 2014c).

**Fig. 4:**
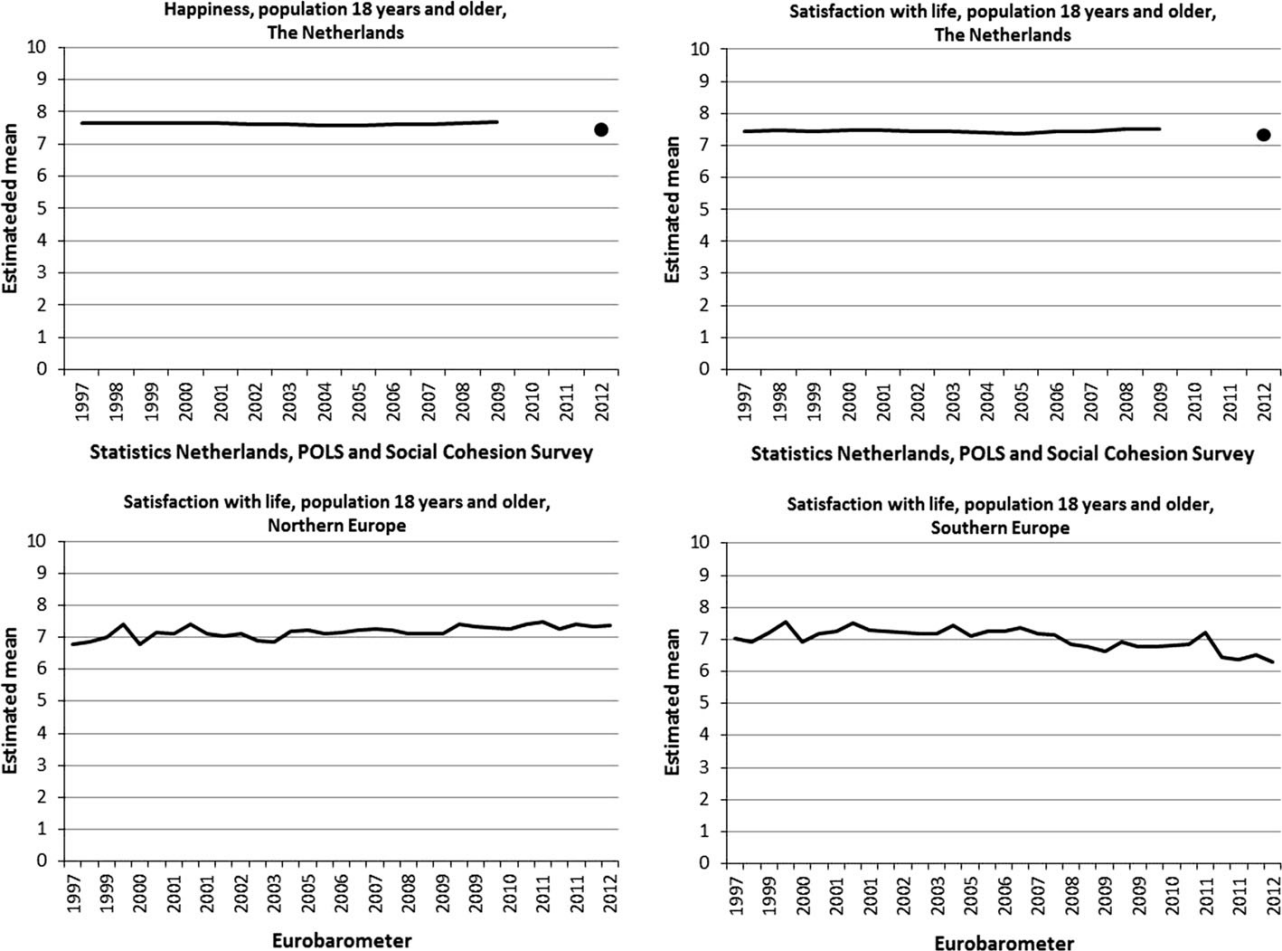
Estimated means happiness and life satisfaction, general population

The application of the Continuum Approach resulted in a best fitting beta distribution for each item and each wave in the corresponding period. We used the estimated parameters of each of these beta distributions to estimate a population mean, the results of which are depicted in Fig. 4.

It is clear from Fig. 4 that there has been little variation in the levels of happiness and life satisfaction over the years in The Netherlands. The average level of life satisfaction in The Netherlands is somewhat lower than that for happiness. In 2012 the levels for both items of Statistics Netherlands dropped. Since no data were collected in 2010 and 2011, it cannot be said whether this is due to a different measurement techniques, or it is an effect of the economic crisis or it has some other cause.

Compared to the flat lines for happiness and life satisfaction based on the surveys of Statistics Netherlands, the time series of estimated Eurobarometer means show a much more fluctuating pattern. Moreover, the time series for Northern Europe shows a slightly upward trend, whereas in Southern Europe the trend is heading downwards.

### Differences in Estimated Means over Time Depending on the Boundaries Used

Just as we did for the general population, we applied the Continuum Approach to the responses for each demographic category for each wave of each item. For each wave we applied the Continuum Approach twice, one time making use of the reference boundaries for the general population and one time making use of the category-specific reference boundaries which are shown in Fig. 3.

As a result we found two best fitting beta distributions for each item and each wave, for which we, just as before, could use the parameters to estimate means on a 0–10 continuum for the corresponding demographic category. As was to be expected and in line with what we presented in Table 1, the estimated means based on the category-specific reference boundaries are not equal to the estimated means based on the reference boundaries for the general population. The differences between the estimated means based on the category-specific boundaries and those based on the boundaries for the entire population however, turn out to be very stable over time. We have summarized this in Table 2, which contains two columns for each item. The first of these columns contains the average difference over the number of waves in the period of observation between the estimated means. The second column contains the standard deviation of these differences from the average difference.

**Table 2:** Average and standard deviation difference in estimated mean

Statistics Netherlands, POLS and Social Cohesion Survey, 1997–2009, 13 waves					
Category	Sub-category	Happiness		Satisfaction with life	
		Difference in estimated mean		Difference in estimated mean	
		Average	Standard deviation	Average	Standard deviation
Sex	Men	0.02	0.01	−0.02	0.01
	Women	−0.02	0.01	0.02	0.01
Age	18–25	−0.07	0.01	−0.08	0.01
	25–35	−0.19	0.01	−0.29	0.01
	35–45	−0.07	0.01	−0.11	0.01
	45–55	0.02	0.01	0.06	0.01
	55–65	0.11	< 0.01	0.20	0.01
	65+	0.11	0.01	0.12	0.01
Edu-cation	Low	0.12	0.01	0.23	0.01
	Middle	0.01	0.01	−0.07	0.01
	High	−0.08	0.01	−0.08	0.02

Eurobarometer 1997–2012, 33 waves					
Category	Sub-category	Satisfaction with life Northern Europe		Satisfaction with life Southern Europe	
		Difference in estimated mean		Difference in estimated mean	
		Average	Standard deviation	Average	Standard deviation
Sex	Men	−0.04	< 0.01	−0.10	< 0.01
	Women	0.03	< 0.01	0.09	< 0.01
Age	18–25	0.06	0.01	0.04	0.01
	25–35	0.21	0.01	0.10	0.01
	35–45	−0.04	< 0.01	0.22	0.01
	45–55	0.12	0.01	−0.09	0.01
	55–65	−0.08	< 0.01	−0.02	< 0.01
	65+	−0.20	0.01	−0.21	0.01
Edu-cation	Low	0.01	0.01	−0.06	0.01
	Middle	0.10	< 0.01	0.08	0.02
	High	−0.01	0.01	−0.12	0.01

**Fig. 5:**
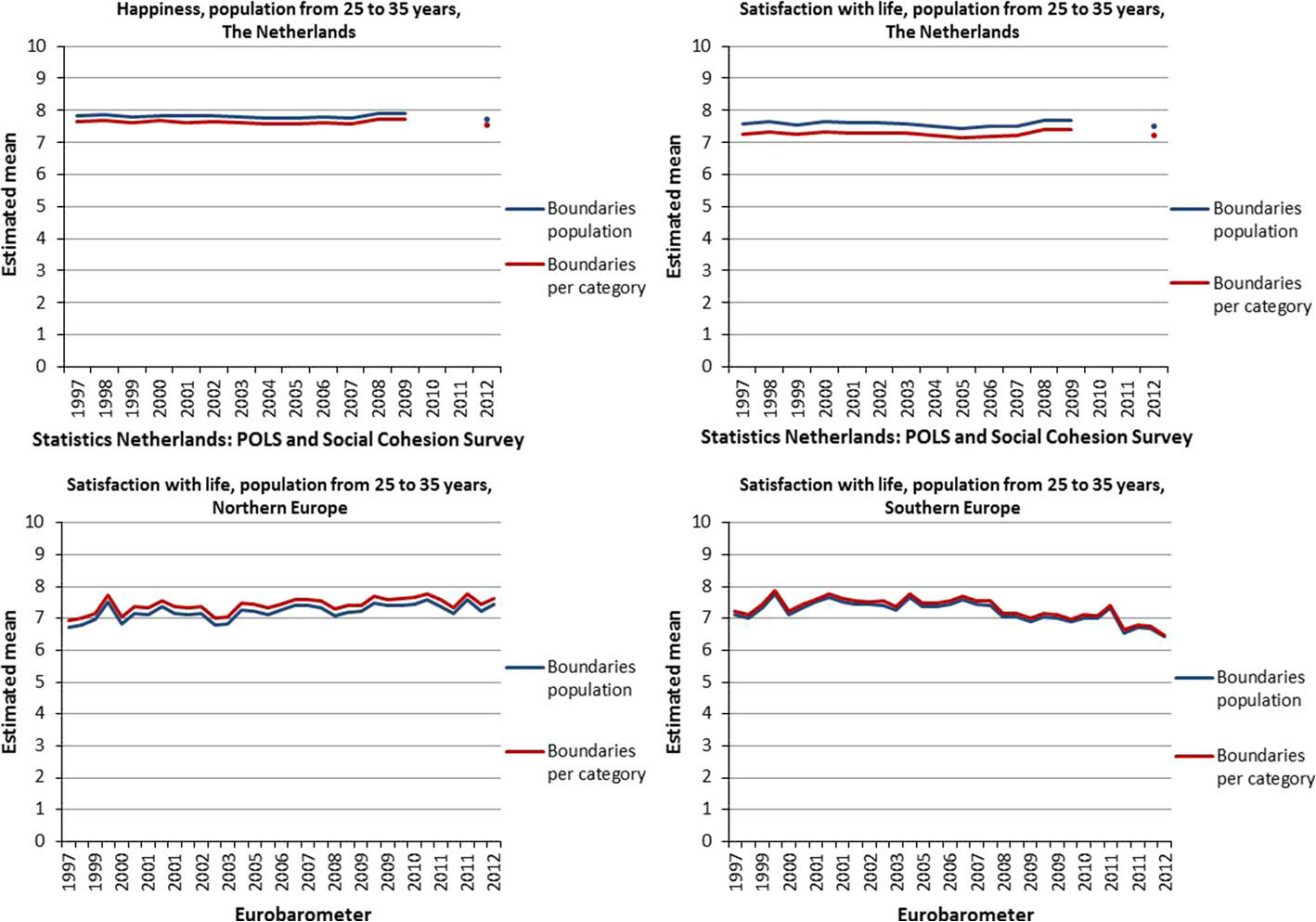
Time series of estimated means among 25–35 years old

The averages in Table 2 are similar to those presented in Table 1. The standard deviations are very small for each demographic category and each item indicating that the differences are rather stable over time. The, in the absolute sense, largest average difference is once more found in the age category 25–35 years for the item of Statistics Netherlands on life satisfaction. A possible explanation for finding the largest difference for this particular category may be that at this stage in their lives individuals in this category must deal with numerous changes, many of which may affect their response to surveys. We have depicted the differences for this demographic category in Fig. 5.

From Fig. 5 it can be seen that for the two items of Statistics Netherlands, the estimated means based on the boundaries for the general population are higher than the estimated means based on the category-specific boundaries, and that this is the opposite for the Eurobarometer item in both Northern Europe and Southern Europe. The patterns of the time series are, however, consistent from which we conclude that if the main interest of a piece of research, as here, is to determine how trends develop then which reference boundaries are used for the conversion becomes less important.

The results shown in Table 2, supported by the visualization in Fig. 5, lead us to conclude that the reference boundaries define the levels of the estimated population means, but do not influence the evolution of the trends. This conclusion makes sense for the flat patterns of the time series for happiness and life satisfaction of Statistics Netherlands, and for the fluctuation patterns for life satisfaction of the Eurobarometer the trend for which go in a different direction in Northern Europe and Southern Europe.

## Discussion

Using three different populations, The Netherlands, Northern Europe and Southern Europe, and items from two surveys, we showed that, from the perspective of monitoring trends in happiness and life satisfaction among demographic categories, it is not necessary to use category-specific reference boundaries to obtain estimated means on a 0–10 continuum if the Continuum Approach is applied. Although we covered some variants for which this conclusion is valid, it would be worthwhile applying the Reference Distribution Method presented in this paper to other topics than happiness and life satisfaction, for example to social cohesion or self-reported health. This would allow us to validate the conclusions presented here more generally.

In Sect. 4.1 we remarked that the levels of the estimated means for the Eurobarometer cannot be compared to those for life satisfaction from the split-half experiment of Statistics Netherlands, due to the different number of response options on the numerical scales used in the surveys. Since we concluded that it is not necessary to use category-specific boundaries to make the transition to the estimated means on a 0–10 continuum, the numerical scale for life satisfaction from the split-half experiment could be used to derive reference boundaries between the response options of the verbal scale of the Eurobarometer item based on the response of the general population measured in 2012. These boundaries in turn could be used to apply the Continuum Approach to the Eurobarometer time series of the different social categories in Northern Europe and Southern Europe. This would enable comparison of trends in life satisfaction among demographic categories in The Netherlands with equivalent categories in Northern Europe and Southern Europe.

The numerical scale from the Eurobarometer was only used in 2011, and as there was no wave of the survey of Statistics Netherlands in this time, this numerical scale from the Eurobarometer cannot be used to apply the Reference Distribution Method to derive reference boundaries for the verbal scale of the life satisfaction item of Statistics Netherlands. There are however, other surveys, such as the European Social Survey, that contain an item on life satisfaction with a numerical scale and that have been fielded over a number of years. The response to such a survey item in a year when both the Eurobarometer and the survey of Statistics Netherlands were fielded could be used substituted to estimate a reference distribution. This reference distribution could then be used accordingly to derive reference boundaries for the general population for the verbal scales of the Eurobarometer item and the Statistics Netherlands item.

In this paper we distinguished between gender, age, and education to differentiate demographic categories of respondents. Another distinction that might have been of interest is that of ethnicity. The conversion of verbal terms into numerical scales is likely to vary between people from a different cultural and linguistic background. The data used for this paper, however, fell short to make this distinction, at least for The Netherlands. We will leave this therefore as a potential direction for future research.

For the trend analyses in this paper we have only focused on mean happiness within demographic categories. We are aware, however, that in practice scholars are often not interested in just the mean, but want to know what the interrelations of the means in subgroups of these demographic groups are, for example from the perspective of inequality or marginalization. Although this falls outside the scope of this paper, we have spent some words on the technique to determine means for subgroups based on a given beta distribution in Appendix 4.

## Main Conclusion

In this paper we addressed the question of whether the transition points derived for the general population can be used for demographic categories to produce reliable extended time series to monitor differences in trends among these categories. We conclude that this is possible and that it is not necessary to derive transition points for each demographic category separately.

## Figures and Tables

**Fig. 6:**
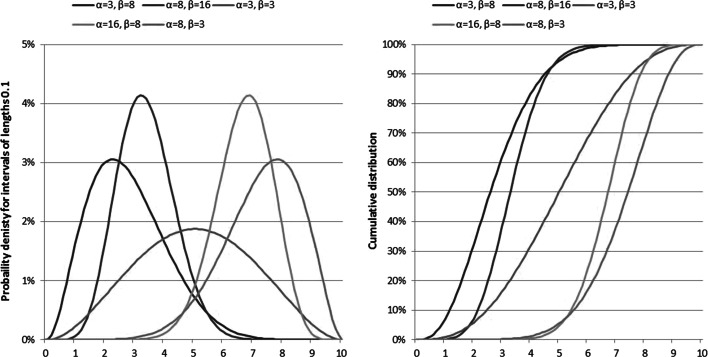
Examples of the probability density β-functions and cumulative distribution β-functions

**Fig. 7:**
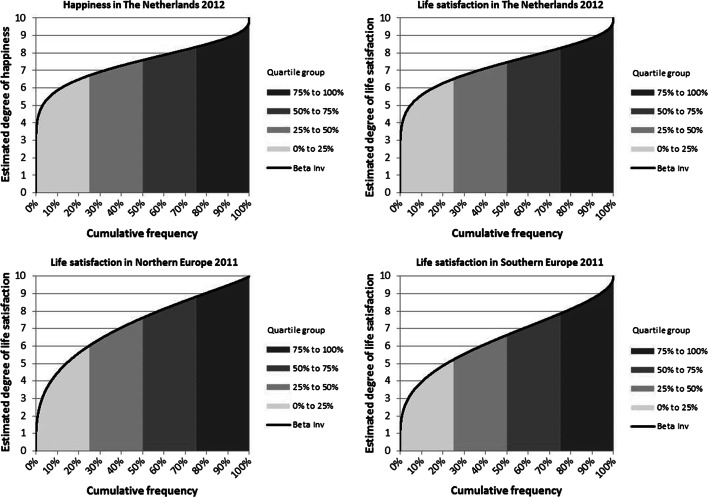
The inverse beta distribution and quartile groups

**Fig. 8:**
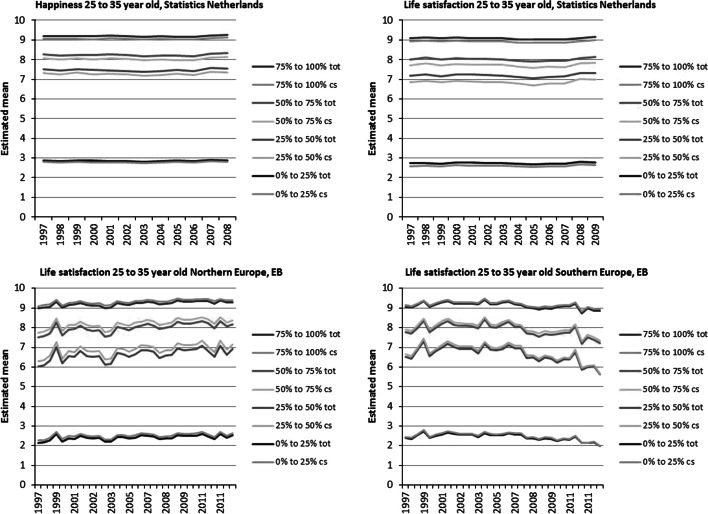
Means of quartile groups of the population aged from 25 to 35 years

## Appendix 1: Frequency Distributions Split-Half Experiment Statistics Netherlands

See Tables 3, 4, 5 and 6.

**Table 3:** Frequency distribution on verbal scale of question ‘To what extent do you consider yourself a happy person?’

Category	Subcategory	N	%DK/NA	Effective N	Unhappy	Not very happy	Neither happy nor unhappy	Happy	Very happy	Total
Total	Total	3,811	0.6	3,787	0.6	3.4	12.7	65.8	17.5	100.0
Sex	Men	1,869	1.7	1,837	0.7	3.6	13.4	64.3	17.9	100.0
	Women	1,974	2.8	1,918	0.5	3.3	11.9	67.2	17.1	100.0
Age	18–25	424	11.6	375	0.9	1.7	9.4	68.0	20.0	100.0
	25–35	566	8.0	521	0.2	1.8	10.1	64.1	23.8	100.0
	35–45	652	0.2	651	0.0	3.7	12.2	64.7	19.5	100.0
	45–55	759	1.2	750	1.0	4.7	13.0	65.2	16.0	100.0
	55–65	706	10.6	631	1.1	4.2	15.8	65.2	13.6	100.0
	65+	798	4.1	765	0.5	3.5	14.0	67.7	14.3	100.0
Education	Low	1,069	3.3	1,034	0.9	4.8	16.6	65.1	12.6	100.0
	Middle	1,619	0.2	1,616	0.5	3.0	11.7	67.7	17.1	100.0
	High	1,105	5.6	1,043	0.2	2.9	9.5	64.0	23.4	100.0

*Source*: Statistics Netherlands, Social Cohesion survey 2012

**Table 4:** Frequency distribution on numerical scale of question ‘To what extent do you consider yourself a happy person?’

Category	Subcategory	N	%DK/NA	Effective N	Completely unhappy	1	2	3	4	5	6	7	8	9	Completely happy	Total
Total	Total	3,701	0.2	3,695	0.3	0.2	0.2	0.6	0.8	3.3	5.7	23.3	43.5	16.1	6.2	100.0
Sex	Men	1,808	1.2	1,787	0.3	0.2	0.2	0.5	0.9	3.0	5.4	22.5	44.3	17.3	5.4	100.0
	Women	1,914	1.5	1,886	0.2	0.1	0.2	0.6	0.7	3.6	5.9	24.0	42.7	15.0	6.9	100.0
Age	18–25	405	11.9	357	0.0	0.2	0.4	0.2	0.9	1.0	6.9	24.6	41.3	16.1	8.3	100.0
	25–35	596	6.5	557	0.0	0.0	0.5	0.5	0.9	3.3	5.0	21.0	45.8	17.5	5.5	100.0
	35–45	664	1.8	652	0.7	0.3	0.0	1.1	0.3	3.3	5.5	22.0	42.1	18.8	5.9	100.0
	45–55	688	1.3	679	0.1	0.1	0.1	0.6	1.4	4.3	5.1	23.7	44.8	14.1	5.6	100.0
	55–65	670	11.6	592	0.5	0.3	0.0	0.2	0.6	5.1	5.6	23.3	43.4	15.6	5.3	100.0
	65+	777	2.3	759	0.2	0.1	0.3	0.5	0.8	2.2	6.3	24.8	42.8	14.9	7.0	100.0
Education	Low	1,064	5.2	1,009	0.9	0.3	0.1	0.9	0.9	4.1	6.4	27.5	39.2	12.5	7.3	100.0
	Middle	1,577	0.9	1,563	0.0	0.0	0.3	0.5	0.8	3.1	6.2	23.3	44.2	15.3	6.4	100.0
	High	1,062	5.5	1,004	0.1	0.0	0.1	0.4	0.5	2.6	4.3	19.0	47.2	21.4	4.3	100.0

*Source*: Statistics Netherlands, Social Cohesion survey 2012

**Table 5:** Frequency distribution on verbal scale of question ‘To what extent are you satisfied with the life you currently lead?’

Category	Subcategory	N	%DK/NA	Effective N	Not very satisfied	Fairly satisfied	Satisfied	Very satisfied	Extraordinarily satisfied	Total
Total	Total	3,819	0.6	3,796	5.9	11.1	44.6	31.2	7.2	100.0
Sex	Men	1,873	1.8	1,840	5.9	11.5	43.5	30.9	8.3	100.0
	Women	1,979	2.8	1,923	6.0	10.7	45.7	31.6	6.1	100.0
Age	18–25	425	11.5	376	5.3	8.6	41.1	37.0	7.9	100.0
	25–35	567	7.9	522	3.3	11.9	37.3	39.5	7.9	100.0
	35–45	654	0.2	653	6.5	10.2	41.1	34.0	8.2	100.0
	45–55	759	1.1	751	7.1	11.4	45.9	28.7	6.8	100.0
	55–65	707	10.6	632	9.9	11.8	45.8	26.8	5.6	100.0
	65+	801	4.1	768	3.2	11.7	52.6	25.6	6.8	100.0
Education	Low	1,071	3.3	1,036	7.9	14.2	50.3	22.9	4.6	100.0
	Middle	1,620	0.1	1,618	5.1	9.6	46.0	32.8	6.4	100.0
	High	1,109	5.6	1,047	5.0	9.7	36.4	38.1	10.9	100.0

*Source*: Statistics Netherlands, Social Cohesion survey 2012

**Table 6:** Frequency distribution on numerical scale of question ‘To what extent are you satisfied with the life you currently lead?’

Category	Subgategory	N	%DK/NA	Effective N	Completely dissatisfied	1	2	3	4	5	6	7	8	9	Completely satisfied	Total
Total	Total	3,694	0.3	3,682	0.3	0.3	0.3	0.9	1.0	4.1	8.0	24.8	39.4	15.1	5.7	100.0
Sex	Men	1,804	1.1	1,785	0.4	0.4	0.4	0.9	1.3	4.0	6.9	23.8	40.4	16.8	4.9	100.0
	Women	1,909	1.6	1,878	0.3	0.2	0.3	0.9	0.7	4.2	9.1	25.8	38.4	13.5	6.5	100.0
Age	18–25	402	11.4	356	0.0	0.0	0.6	0.9	0.5	4.3	7.9	26.3	37.3	14.4	7.9	100.0
	25–35	592	6.3	555	0.0	0.5	0.2	1.2	2.1	3.4	9.9	26.4	37.5	14.7	4.1	100.0
	35–45	662	1.7	651	0.7	0.3	0.0	1.3	0.9	5.5	7.3	24.9	37.2	16.6	5.5	100.0
	45–55	686	1.5	676	0.1	0.3	0.7	0.5	1.5	3.9	8.2	27.3	36.9	15.0	5.6	100.0
	55–65	671	11.6	593	1.0	0.0	0.4	0.8	0.3	4.5	7.3	22.6	42.9	15.1	5.2	100.0
	65+	775	2.5	756	0.2	0.3	0.2	0.8	0.6	3.2	7.6	22.4	43.4	14.8	6.5	100.0
Education	Low	1,063	5.0	1,010	1.1	0.5	0.2	1.6	1.2	4.4	9.1	24.9	36.6	12.7	7.8	100.0
	Middle	1,574	1.1	1,557	0.0	0.1	0.5	0.9	0.8	4.8	8.4	25.3	40.8	13.4	5.0	100.0
	High	1,058	5.6	999	0.1	0.1	0.2	0.1	0.9	2.5	6.4	24.5	40.1	20.6	4.5	100.0

*Source*: Statistics Netherlands, Social Cohesion survey 2012

## Appendix 2: Frequency Distributions Eurobarometer 76.3 2011 and 76.2 2011

See Tables 7, 8, 9 and 10.

**Table 7:** Frequency distribution Northern Europe on verbal scale of question ‘On the whole how satisfied are you with the life you lead?’

Category	Subcategory	N	%DK/NA	Effective N	Not at all satisfied	Not very satisfied	Fairly satisfied	Very satisfied	Total
Total	Total	9,940	0.3	9,914	2.9	11.2	56.0	29.9	100.0
Sex	Men	5,769	0.2	4,759	2.9	10.5	56.9	29.7	100.0
	Women	5,171	0.3	5,155	2.9	11.8	55.1	30.2	100.0
Age	18–25	1,123	0.2	1,121	1.7	9.4	51.3	37.6	100.0
	25–35	1,520	0.1	1,519	3.3	12.2	56.7	27.7	100.0
	35–45	1,715	0.1	1,713	2.6	12.6	57.1	27.7	100.0
	45–55	1,830	0.1	1,828	4.2	13.5	57.5	24.8	100.0
	55–65	1,514	0.1	1,512	3.6	11.0	54.2	31.2	100.0
	65+	2,237	0.8	2,219	2.0	8.4	56.9	32.7	100.0
Education	Low	1,771	0.6	1,761	3.8	15.2	57.5	23.5	100.0
	Middle	4,252	0.2	4,244	3.9	13.1	55.6	27.3	100.0
	High	3,165	0.1	3,162	1.6	7.4	55.9	35.1	100.0

*Source*: Eurobarometer 76.3 2011

**Table 8:** Frequency distribution Northern Europe on numerical scale of question ‘On the whole how satisfied are you with the life you lead?’

Category	Subcategory	N	%DK/NA	Effective N	Very dissatisfied	2	3	4	5	6	7	8	9	Very satisfied	Total
Total	Total	9,989	0.2	9,972	0.8	1.2	2.0	1.7	7.4	6.4	14.8	27.5	18.0	20.2	100.0
Sex	Men	4,840	0.1	4,833	0.9	1.1	2.2	1.8	6.6	6.4	14.9	28.1	19.3	18.7	100.0
	Women	5,149	0.2	5,139	0.7	1.3	1.8	1.7	8.1	6.4	14.7	27.0	16.7	21.7	100.0
Age	18–25	1,135	0.4	1,131	0.7	1.1	1.4	0.8	3.9	4.5	14.8	25.0	20.1	27.8	100.0
	25–35	1,511	0.1	1,510	0.9	1.1	2.6	1.5	5.9	4.8	15.9	25.2	21.3	21.0	100.0
	35–45	1,725	0.1	1,724	1.3	1.3	2.1	1.3	7.8	5.4	16.1	29.7	17.7	17.2	100.0
	45–55	1,853	0.2	1,849	0.5	1.7	1.6	2.3	9.2	8.7	13.0	29.1	16.5	17.3	100.0
	55–65	1,519	0.3	1,514	1.3	1.6	2.2	2.0	6.9	6.5	14.3	26.5	19.0	19.7	100.0
	65+	2,248	0.1	2,245	0.4	0.5	2.0	1.9	8.7	7.3	14.7	28.0	15.4	21.0	100.0
Education	Low	1,869	0.2	1,866	1.1	1.9	2.6	3.1	10.6	8.6	14.6	25.8	13.0	18.8	100.0
	Middle	4,356	0.1	4,353	1.0	1.4	2.2	1.4	8.8	6.8	15.2	26.9	16.4	19.8	100.0
	High	3,088	0.4	3,077	0.5	0.5	1.5	1.2	4.0	4.6	13.9	30.1	23.0	20.9	100.0

*Source*: Eurobarometer 76.2 2011

**Table 9:** Frequency distribution Southern Europe on verbal scale of question ‘On the whole how satisfied are you with the life you lead?’

Category	Subcategory	N	%DK/NA	Effective N	Not at all satisfied	Not very satisfied	Fairly satisfied	Very satisfied	Total
Total	Total	5,037	0.6	5,009	9.1	28.0	57.3	5.6	100.0
Sex	Men	2,426	0.7	2,408	8.4	26.7	58.8	6.0	100.0
	Women	2,611	0.4	2,601	9.7	29.2	55.9	5.2	100.0
Age	18–25	508	0.8	504	6.3	24.4	61.1	8.1	100.0
	25–35	826	0.6	821	7.1	30.3	56.0	6.6	100.0
	35–45	1,059	0.4	1,055	9.2	30.1	54.7	6.0	100.0
	45–55	814	0.0	814	8.7	27.8	57.7	5.8	100.0
	55–65	705	0.3	703	10.0	27.0	57.5	5.5	100.0
	65+	1,123	1.1	1,111	11.3	26.8	58.7	3.2	100.0
Education	Low	1,757	0.5	1,748	12.5	30.4	53.6	3.5	100.0
	Middle	1,807	0.3	1,802	7.7	28.5	57.5	6.2	100.0
	High	968	0.2	966	5.5	21.2	64.7	8.6	100.0

*Source*: Eurobarometer 76.3 2011

**Table 10:** Frequency distribution Southern Europe on numerical scale of question ‘On the whole how satisfied are you with the life you lead?’

Category	Subgategory	N	%DK/NA	Effective N	Very dissatisfied	2	3	4	5	6	7	8	9	Very satisfied	Total
Total	Total	5,088	0.1	5,084	0.9	1.1	2.3	3.9	10.4	15.5	22.6	26.3	10.2	6.9	100.0
Sex	Men	2,464	0.1	2,462	0.7	1.5	2.6	4.1	10.4	15.4	22.0	27.3	9.7	6.5	100.0
	Women	2,624	0.1	2,622	1.1	0.8	2.1	3.8	10.4	15.6	23.1	25.2	10.7	7.2	100.0
Age	18–25	534	0.0	534	0.2	1.3	0.4	3.4	8.2	14.0	20.8	28.3	13.7	9.7	100.0
	25–35	809	0.0	809	1.2	0.7	2.1	4.1	8.2	13.8	20.9	26.0	15.3	7.7	100.0
	35–45	1,020	0.0	1,020	1.5	0.9	1.9	1.9	9.2	14.3	22.1	28.1	11.2	9.0	100.0
	45–55	882	0.0	,882	0.3	1.6	2.9	3.5	11.2	13.5	24.8	28.1	8.3	5.7	100.0
	55–65	709	0.0	,709	1.0	1.0	2.3	5.1	9.6	14.0	26.1	26.0	7.9	7.2	100.0
	65+	1,129	0.4	1,125	0.9	1.3	3.2	5.3	14.0	21.0	21.2	22.6	6.8	3.6	100.0
Education	Low	1,793	0.1	1,791	1.5	1.5	3.5	5.7	12.8	18.1	22.5	22.1	7.0	5.2	100.0
	Middle	1,721	0.0	1,721	0.3	0.6	1.2	3.5	9.3	15.6	24.5	27.1	9.9	8.0	100.0
	High	1,093	0.0	1,093	0.6	1.3	2.6	2.7	6.9	11.0	20.5	31.5	15.1	8.0	100.0

*Source*: Eurobarometer 76.2 2011

## Appendix 3: Outline of the Continuum Approach

The main assumption of the Continuum Approach is that variables are continuously distributed in a population and in the case of happiness as variable can be described by a beta distribution on a continuum from 0 to 10. The family of beta distributions consists of a series of distributions that are each characterized by two shape parameters, α and *β,* that are positive real numbers.

A beta distribution can be expressed using the complete beta function:

1 $$B\left( {\alpha ,\beta } \right) : = \mathop \smallint \limits_{0}^{1} t^{\alpha - 1} \left( {1 - t} \right)^{\beta - 1} dt$$

Given the formula (Eq. 1) the probability density function of the beta distribution on the continuum from 0 to 10 can be written as:

2 $$f\left( {x|\alpha ,\beta } \right)\text{ := }\left\{ {\begin{array}{*{20}c} {\left[ {10B\left( {\alpha ,\beta } \right)} \right]^{ - 1} x^{\alpha - 1} \left( {10 - x} \right)^{\beta - 1} } & {{\text{for}} \, x \in \left[ {0,10} \right]} \\ 0 & {\text{otherwise}} \\ \end{array} } \right.$$

The mean *μ* and standard deviation *σ* of a beta distribution with parameters α and *β* on the continuum from 0 to 10 are equal to:

3 $$\upmu = 10\frac{\alpha }{\alpha + \beta }$$

4 $$\sigma = 10\sqrt {\frac{\alpha \beta }{{\left( {\alpha + \beta } \right)^{2} \left( {\alpha + \beta + 1} \right)}}}$$

To make this less abstract we give some examples of the probability density functions and the cumulative distribution functions for different values of *α* and *β* in Fig. 6. If *α* < *β*, the probability density function is skewed to the right, if *α* > *β* the function is skewed to the left and if both parameters are equal the function is symmetric about *x* = 5. Furthermore, the larger the values of *α* and *β*, the more peaked the density curve and the steeper the cumulative distribution curve are.

A starting point for the Continuum Approach to happiness is provided by the cumulative frequencies of measured happiness on a discrete primary scale and the values on the continuum from 0 to 10 at which respondents change their judgment from one to the adjacent response option on this primary scale, for example from ‘happy’ to ‘very happy’. On basis of the cumulative frequencies and the values on the continuum of the boundaries between the response options of the primary scale, the shape parameters *α* and *β* of the best fitting beta distribution are estimated in the Continuum Approach as maximum likelihood estimators. This estimation procedure is described into more detail in Kalmijn (2010; pp. 160–162). There is always a perfect fit in the case of a primary scale with three response options. If the number of response options is restricted to only two, then there is no single solution: the number of perfectly fitting beta distributions is infinite and the application of the Continuum Approach is useless. In the case of at least four response options, then in general there will be no perfectly fitting beta distribution and the best fitting solution should be taken. Those who are interested in the methodological considerations of the Continuum Approach can find more information about it in Kalmijn (2010, Ch. VI) and Kalmijn et al. (2011).

## Appendix 4: Means for Subgroups Derived from a Beta Distribution

Suppose that the parameters *α* and *β* of the estimated beta distribution for happiness or life satisfaction in a population are given. The mean happiness or life satisfaction within subgroups of this population can accordingly be estimated by making use of the inverse beta distribution.^7^ In Fig. 1 we have depicted the beta distribution for life satisfaction in The Netherlands in 2012. The parameters of this distribution are: *α* = 8.31 and *β* = 3.05. The value of the inverse of this beta distribution for the 25th percentile point is equal to 6.5, meaning that the estimated value of life satisfaction is 6.5 or lower for the subgroup of the 25 % least satisfied people in the population. In a similar way the inverse of the beta distribution at the 75th percentile point is equal to 8.3, meaning that the value of life satisfaction for the subgroup of the 25 % most satisfied people is at least 8.3. The inverse beta distributions of the reference distributions derived from the numerical scales of the split-half experiment of Statistics Netherlands and the Eurobarometer are shown in Fig. 7. In each graph of this figure we have also depicted four quartile groups. The most left of the quartile groups represents the least happy or satisfied people in the population and the most right quartile group represents the most happy or satisfied people.

It can, for example, be seen in Fig. 7 that the degree of life satisfaction in 2012 within the second quartile group in The Netherlands varies from 6.5 to 7.5. In the corresponding subgroup in Southern Europe the degree of life satisfaction varies from 5.2 to 6.6.

We calculated the value of the inverse beta distribution for 25.000 equidistant points in each quartile group. The average of this large number of values for a quartile group is an estimate of the mean happiness or life satisfaction in this group. The estimates of the mean life satisfaction in The Netherlands calculated in this way for the four quartile groups are equal to respectively 5.6, 7.0, 7.9 and 8.8. We have estimated the means for the quartile groups of all the demographic categories we have distinguished in this paper, based on both the beta distribution estimated using category-specific reference boundaries and by using the reference boundaries for the general population. We did not find noteworthy differences in the evolution of the trends depending on the reference boundaries used. This is illustrated for the age group from 25 to 35 years old in Fig. 8. We used the abbreviation ‘cs’ to denote the use of the category-specific boundaries to estimate the means and the abbreviation ‘tot’ to denote the use of the boundaries for the general population to estimate the means for the boundaries.

## Footnotes

1 We use the term ‘anchor points’ for the response options at both ends of a discrete scale. In the case of a continuous scale, we use the term ‘extremes’ to refer to the boundaries of the scale.

2 Throughout this paper we use the term item for a leading question and its corresponding response options.

3 Happiness: http://statline.cbs.nl/StatWeb/publication/?DM=SLEN&PA=60027ENG&D1=43-47&D2=1-2,12,15,19-22,29-31&D3=a&LA=EN&VW=T

4 Satisfaction with life: http://statline.cbs.nl/StatWeb/publication/?DM=SLEN&PA=60027ENG&D1=48-52&D2=1-2,12,15,19-22,29-31&D3=a&LA=EN&HDR=T&STB=G1,G2&VW=T

5 Permanent Survey on Living Conditions.

6 The boundaries between the response options of a 10-point numerical scale are positioned at different values than those between the response options of an 11-point numerical scale.
